# Metformin and Covid-19: Focused Review of Mechanisms and Current Literature Suggesting Benefit

**DOI:** 10.3389/fendo.2021.587801

**Published:** 2021-07-22

**Authors:** Sherehan Ibrahim, Jamie R. Lowe, Carolyn T. Bramante, Surbhi Shah, Nichole R. Klatt, Nancy Sherwood, Louis Aronne, Michael Puskarich, Leonardo Tamariz, Ana Palacio, Eric Bomberg, Michael Usher, Samantha King, Brad Benson, Deneen Vojta, Chris Tignanelli, Nicholas Ingraham

**Affiliations:** ^1^ Department of Medicine, Division of General Internal Medicine, University of Minnesota, Minneapolis, MN, United States; ^2^ MPH Program, Dartmouth College, Hanover, NH, United States; ^3^ Department of Medicine, Division of Hematology, Oncology and Transplant, University of Minnesota, Minneapolis, MN, United States; ^4^ Department of Surgery, University of Minnesota, Minneapolis, MN, United States; ^5^ Department of Epidemiology, University of Minnesota, Minneapolis, MN, United States; ^6^ Division of Endocrinology, Cornell Weill College of Medicine, New York, NY, United States; ^7^ Department of Emergency Medicine, University of Minnesota, Minneapolis, MN, United States; ^8^ Division of Cardiology and Miami VA Healthcare Administration, University of Miami, Miami, FL, United States; ^9^ Department of Pediatrics, Division of Pediatric Endocrinology, University of Minnesota, Minneapolis, MN, United States; ^10^ UnitedHealth Group, Research and Development, Plymouth, MN, United States; ^11^ Department of Medicine, Division of Pulmonary, Allergy, Critical Care, and Sleep Medicine, University of Minnesota, Minneapolis, MN, United States

**Keywords:** metformin, COVID-19, mechanisms of action, obesity, microbiome

## Abstract

Metformin is the first-line medication for type 2 diabetes, but it also has a long history of improved outcomes in infectious diseases, such as influenza, hepatitis C, and *in-vitro* assays of zika. In the current Covid-19 pandemic, which has rapidly spread throughout the world, 4 observational studies have been published showing reduced mortality among individuals with home metformin use. There are several potential overlapping mechanisms by which metformin may reduce mortality from Covid-19. Metformin’s past anti-infectious benefits have been both against the infectious agent directly, as well as by improving the underlying health of the human host. It is unknown if the lower mortality suggested by observational studies in patients infected with Covid-19 who are on home metformin is due to direct activity against the virus itself, improved host substrate, or both.

## Introduction

Metformin was discovered in the 1920s (1). In the 1940s-50s, metformin showed benefit when used in influenza infection, and was noted to lower glucose, but not below physiologic levels (2). Other biguanide medications then had safety issues, so by association metformin then fell out of favor until the 1990s (1). Currently, metformin has Food and Drug Administration (FDA) approval as a first-line medication for type 2 diabetes, on and off-label indications for diabetes prevention in prediabetes; and off label for polycystic ovarian syndrome; anti-psychotic associated weight gain; weight loss; gestational diabetes; and fertility enhancement (3, 4). There is also mounting evidence supporting the potential effects of metformin beyond glucose control in the aging population (5). It appears that increased biologic aging may be a key underlying risk factor for poor outcomes from severe acute respiratory syndrome coronavirus 2 (SARS-CoV-2) disease, Covid-19, as Covid-19 has disproportionately affected older individuals (6, 7).

Several observational cohort studies have been published in peer review journals showing associations with reduced mortality from Covid-19 among patients already who were already on metformin (**Table 1** **)** (8, 9, 11–13). It appears that metformin may be associated with less severe Covid-19 disease, however there are no prospective studies published to date. It must be highlighted that observational results are not conclusive because of the inherent challenge of eliminating residual confounding. Comorbidities associated with Covid-19 outcomes (i.e. hypertension, coronary artery disease, obesity) are commonly present in groups exposed and not exposed to metformin in retrospective cohorts, which make it challenging to know if observational data would be replicated in randomized, prospective trials (7, 28).

**Table 1:** Overview of papers in 2020 with findings related to metformin and Covid-19.

Author	Population	Methods		Finding	Mechanism or other findings
**Outcomes in Covid-19**					
Luo et al. (8) *Am J Trop Med Hyg*	283 adults with T2DM hospitalized with Covid-19 in Wuhan.		Retrospective cohort, 104 adults on metformin with 179 not on metformin. Metformin use appears to be home metformin use.	- Hospital mortality, metformin *vs* no metformin: 2.9% *vs* 12.3% (p=0.01)- No difference in length of stay- No associations with other T2DM meds- OR for survival: 4.36 (1.22-15.59, p=0.02)	Lower glucose levels in the metformin group, 9.19 *vs* 7.36 (p<0.01), no difference in neutrophils or lymphocytes.	
Cariou et al (9), “Coronado study” *Diabetalgia*	1,317 adults with T2DM in France, with or without home metformin use		Multi-center observational study. Main outcome: mortality or intubation; Secondary outcome was mortality	- HbA1C not associated with main outcome (p=0.28) or death (p=0.91) - Preadmission metformin use associated with lower mortality OR 0.59 (0.42, 0.84),- No association with other T2DM meds - OR mortality with insulin 1.71 (1.20, 2.43)	CRP 1.99, (1.69, 2.43), lymphocyte count (OR 0.69, 0.60-0.80), fibrinogen OR 1.32 (1.09, 1.58) and AST (OR 2.23, 1.70-2.93) predicted mortality.	
Crouse et al. (10), *Frontiers in Endocrinology*	25,326 subjects tested for Covid-19 between 2/25/20 and 6/22/20 in Alabama.		Retrospective electronic health records study assessing mortality in Covid-19	- Association between prior metformin use and a reduction in mortality (OR 0.33, 95% CI 0.13-0.84; p=0.02) compared to those with T2DM not on metformin.	Glucose levels similar between both groups, Metformin mechanism may reside outside of its glycemia.	
Bramante et al (11), *Lancet Health and Longevity*	6,256 adults with T2DM or obesity hospitalized for Covid-19 in the US		Retrospective review of USA UnitedHealth Group claims data; 2,333 in metformin group, 3,923 in no-metformin group	- Metformin associated with reduced mortality in females: OR 0.759 (0.601, 0.960) by propensity matching; OR 0.780 (0.631, 0.965) by mixed effects; OR 0.785 (0.650, 0.951) by Cox proportionalhazards.	In same sample, TNFα inhibitors were associated with decreased mortality (only 38 patients), suggesting TNFα a possible pathway.	
Lalau et al (12), *Diabetes & Metabolism*	2449 adults with T2DM with or without previous metformin use		Multi-center observational study. Main outcome: mortality or intubation within 7 days and 28 days of admission	- Mortality rate in metformin users *vs* non: day 7 (8.2 *vs* 16.1%, *P* < 0.0001); day 28 (16.0 *vs* 28.6%, *P* < 0.0001)- Mortality by propensity score weighting, metformin users *vs* non: day 7 OR 0.67 (0.47−1.01); day 28 0.71 (0.54−0.94).	Metformin users presented greater case severity on admission regarding clinical, radiological, and biological features, compared with non-users.	
Lukito et al (13), *Diabetes & Metab Synd: Clin Res& Rev*	Meta-analysis of 10,233 adults across 9 studies		The mean NOS of the included studies was 8.55 ± 0.52, indicating high-quality studies.	- Metformin use associated with lower mortality in pooled non-adjusted model, OR 0.45 (0.25, 0.81), p=0.008; and adjusted (OR 0.64 (0.43, 0.97), p = 0.035.	SARS-CoV-2 damages β-cells. Optimal control of T2DM, for chronic and transient cases, may help in treating COVID-19	
**Mechanistic, diabetes, and safety findings in Covid-19**					
Chen et al. (14) *Diabetes Care*	904 patients with Covid-19, 136 of whom had T2DM	Characteristics and outcomes of patients with T2DM and Covid-19. No results reporte for use of GLP-1 receptor agonists.		- Metformin users *vs* non-users: No significant difference in likelihood of ‘poor prognosis’: 30% *vs*. 50%, p=0.688- In PCR-confirmed cases, no difference in in-hospital death (18.2% *vs* 26.1%, p=0.77). No associations with DDP-4i’s	Metformin users had lower IL-6 (4.07 *vs* 11.1, p=0.02). In PCR-confirmed cases, IL-6 was also lower in metformin users than non-metformin users (4.77 *vs* 11.1, p=0.024).
Zhu et al. (15) *Cell metabolism*	952 adults with T2DM Covid-19 and in Hubei, China	Retrospective review. Metformin was given in hospital to 278 patients.		- Metformin was more likely to be given to those with poor glucose control.- No metformin specific results reported.	Worse glucose control associated with mortality and end-organ complications.
Montastruc et al. (16)	10,771 ICSRs involving hydroxychloroquine	Retrospective review, outcomes of mortality		- Hydroxychloroquine + metformin associated with a ROR of 57.7 (23.9-139.3) compared to hydroxychloroquine- Hydroxychloroquine + metformin was associated with a ROR value of 6.0 (2.6-13.8) compared to metformin alone	More autophagosomes in heart, liver, kidneys of mice treated with both. Synergism of inhibition of mitochondrial complex I, and autophagy from hydroxychloroquine (17).
Huh et al (18), medrxiv.org	65,149 adults,claims data in S Korea.	Case control study, metformin (n=219) *vs* control (n=3604)		- Risk of Infection, crude OR 0.69 (0.60-0.80), aOR: 0.95 (0.81-1.11).	Covariates: sex, age, region, comorbidities, meds, utilization
Nafakhi et al, *Diabetes & Metab Synd: Clin Research & Reviews*	192 patients with COVID-19 pneumonia, of whom 67 patients had T2DM	Retrospective cohort of patients with newly diagnosed COVID-19 pneumonia; August 20, to October 5, 2020 in Iraq		- Metformin use associated with lower ICU days, OR 0.30 (0.20-0.40, p=0.03); hospital days, OR 0.40 (0.20-0.30,p=0.02); and in-hospital mortality OR 0.10 (0.1–0.6), p = .025.	Insulin use was associated with extensive lung injury and post-acute COVID-19 pneumonia partial recovery
Cheng et al. (19)	1,213 patients with Covid-19 and T2DM	Retrospective cohort of individuals hospitalized		HR Acidosis 2.73 (1.04-7.13,p=0.04); lactic acidosis 4.46 (1.11,18.00,p=0.04)	Appears to be for use of 2-3g/day during hospitalization.
**Evidence that metformin does reduce TNF-alpha in both males and females.**					
Author	Population			Finding	
Krysiak et al. (20)	Humans, 36% female, did not compare men *vs* women.			After 12 weeks of treatment, metformin “reduced plasma C-reactive protein levels and monocyte release of TNF*α* and IL-6, as well as tended to reduce monocyte release of IL-1*β *and monocyte chemoattractant protein-1, which was accompanied by an improvement in insulin sensitivity.	
Andrews et al. (21)	Humans, men only with obesity and diabetes, ave age 55 years.			Those “treated with metformin had lower levels of hsCRP expression of TNFα and TLR 2/4, than their counterparts not receiving the drug.”	
Hyun et al. (22)	Mice, male only			Metformin suppresses scavenger receptors in macrophages, down-regulates TNFα**	
**Metformin and sex-specific findings.**					
Author	Population			Finding	
Park, J, et al. (23)	Patients with colorectal cancer			Interaction test between metformin and sex after adjustment for relevant factors revealed that female CRC patients taking metformin exhibited a significantly lower CRC-specific mortality rate than male CRC patients taking metformin (HR = 0.369, 95%CI: 0.155-0.881, *P* = 0.025). Subgroup analysis revealed significant differences in CRC-specific mortality between the metformin and non-metformin groups in female patients (HR = 0.501, 95%CI: 0.286-0.879, *P* = 0.013) but not male patients (HR = 0.848, 95%CI: 0.594-1.211, *P* = 0.365).	
DPP (24 **)**	Adults with overweight & preDM			Metformin reduced CRP by 7% in men and 14% in women	
Quan, H, et al. (25)	105 human patients			Combined exenatide and metformin showed better effects on female than male patients for improving insulin sensitivity and serum lipid profile, reducing insulin resistance, increasing adiponectin levels, and decreasing the levels of HbA1c, BMI, resistin, TNF-alpha, CRP (p<0.05).	
Naffaa et al (26),	113, 749 patients who started metformin from 1998-2014.			Adherence assessed by the mean proportion of follow-up days covered (PDC) with metformin. Adherence with was associated with a reduced risk of developing RA in women, not men.	
Jiang et al. (27)	328 patients with T2D and Covid, 100 of which were on metformin while hospitalized			In the mixed-effected model, metformin use was associated with the lower incidence of ARDS.	
				Metformin may have potential benefits in reducing the incidence of ARDS in patients with COVID-19 and type 2 diabetes. However, this benefit differs significantly by gender as confirmed by subgroup analysis, metformin use was associated with the lower incidence of ARDS in females.	

T2DM, Type 2 diabetes mellitus; PCR, Polymerase chain reaction; GLP-1, glucadon-like-peptide 1; DDP-4, Dipeptidyl peptidase-4; WBC, White blood cells, HbA1C, Hemoglobin A1c; CRP, C-reactive protein, AST, Aspartate aminotransferase; OR, odds ratio; ROR, Risk Odd Ratio; AMPK, adenosine monophosphate protein kinase; mTOR, mammalian target of rapamycin; NFK, nuclear factor kappa light enhancer of activated B cells (NFKB); TLR, Toll Like Receptor. DPP, Diabetes Prevention Research Group; CRC, Colorectal cancer; BMI, Body mass index; RA, Rheumatoid arthritis.

Here, we present a targeted review of the literature on the mechanistic reasons why metformin might improve outcomes from Covid-19, and a review of the observational data showing the potential benefit from metformin use in Covid-19. Furthermore, given the known sex-specific differences in Covid-19 outcomes (29) and sex-specific associations between metformin and mortality from Covid-19, we also highlight potential sex-specific effects of metformin in this review.

## Primary Mechanism of Actions

Currently and prior to the Covid-19 pandemic, the molecular mechanisms by which metformin works have been a topic of debate and responses to metformin have often been variable (30, 31). In patients with diabetes mellitus, metformin improves glycemia largely by decreasing hepatic gluconeogenesis, primarily through phosphorylation of AMP-activated protein kinase (AMPK) (32). Activation of AMPK enhances hepatic insulin sensitivity and gut utilization of glucose, promoting glucagon-like peptide-1 (GLP-1) secretion, and favorably altering the gut microbiome (33). Metformin has been found to have cytokine-reducing effects in both patients with and without diabetes mellitus, which may be of primary import in Covid-19 (34).

The effect of metformin on cytokines has been thought to be mediated by blocking the AMPK cytokine receptor pathway and thus decreasing in proinflammatory genes, though some literature has suggested AMPK-independent means exist as well (34–36).

Cytokine storm leads to severe disease in COVID 19, and hence contributes significantly to morbidity and mortality (37). By the virtue of dampening the cytokine storm, metformin may reduce the morbidity in COVID 19. The many possible mechanisms by which metformin may be protective against Covid-19 have been the topic of several recent papers (38–41). A summary of mechanisms by which metformin has been theorized to convey benefit from Covid-19 is found in **Table 2**. We also summarize published literature evaluating the relationship between Covid-19 and metformin use.

Table 2Overview of mechanisms of action of metformin and their relationship to SARS-CoV-2 infection.

**Table:** 

Pathways	Metformin’s overlapping mechanisms of action	Theorized Covid-19 relationship
I. Viral entry and lifecycle	Activates AMPK, which can lead to conformational changes to ACE2 (39, 42, 43). inhibits mTOR reducing -viral protein complexes central to viral replication (39).	Decreased SARS-CoV-2 entry *via* the ACE2, and replication through Orf9c and Nsp7 (38, 39, 42, 43).
II. Immune modulation includes	Decreases IL-6, TNF*α*, and suppresses c-c motif chemokine ligand (34, 44–46); Inhibits TLR-7 signaling (40, 47, 48). Possibly boosts IL-10 as well (IL-10 is hard to interpret because it may be elevated in a response to reduce TNF*α*.) (34)	These cytokines contribute to morbidity in Covid-19 (37, 49, 50). Chen et al. found lower IL-6 with metformin use (14).
III. Neutrophil-extracellular traps	Decreases neutrophil-extracellular traps (NETs), which are released from neutrophils and contain DNA, histones, and proteins that are microbiocidal (51, 52). Neutrophil count dropped by >1,000 cells/mm (3) in 3 months of metformin (53). Patients with Covid-19 have had elevated levels of histones and DNA components from NETs (54). A byproduct of NETs may accelerate viral entry (55)	Excessive NET formation leads to cytokine storm and microthrombus (possibly independent of tissue factor), and ARDS in Covid-19 (54). Neutrophil infiltration in pulmonary capillaries have been an important feature of severe Covid disease (7, 34, 56, 57).
IV. Decreased glycemia	Phosphorylates AMPK (32), improving hepatic insulin sensitivity, gut utilization of glucose, GLP-1 secretion and favorably altering the gut microbiome (33).	Glycemia is associated with increased length of stay and mortality in patients with Covid-19 (58).
V. Mast cell stabilization	Inhibits IgE- and aryl hydrocarbon- mediated mast cell activation (59). Mast cells in female rats cause greater increase in TNF*α* than mast cells in male rats, which may explain a larger benefit from metformin in women than men with Covid (60).	Mast cell activation has been cited as an early indicator of inflammatory response to SARS-CoV2 and cytokine storm (61, 62).
VI. Decreased thrombosis	Decreases thrombosis in longterm follow-up, felt to be by inhibiting platelet activation factor and mtDNA release (63, 64).	Thrombosis is an important component of Covid-19 pathology (65, 66).
VII. Endothelial function	Significantly decreases HOMA-IR and non-significant decrease in tissue plasminogen activating factor 8 weeks after randomization to metformin (67).	Pulmonary vascular endothelitis has been found in lungs of patients with Covid-19 (68).
VIII. Pulmonary fibrosis	Increase resolution of fibrosis *via* AMPK activation of lung myofibroblasts (69) Reduced pulmonary fibrosis (through NFK, reduced TGF-beta, VEGF) (41).	Fibrosis occurs after Covid-infection, especially in patients with high IL-6 (70).
IX. Endosomal pH	Increasing pH *via* action on vacuolar ATPase, endosomal Na+/H+ exchangers (71)	High endosomal pH inhibits viral replication.

**Table:** 

Proposed assays to assess these pathways in patients with Covid-19, with and without metformin use:	
- Flow cytometry; mean platelet volume	- IFABP and LPS limulus assay to assess gut-epithelial integrity and microbiotal translocation
- ELISA assays for glycemia and inflammatory markers	- M30-apoptosis ELISA (for CK-18) to assess the influence of hepatic steatosis
- MPO/dsDNA and citrullinated-histone H3 to assess NETs	- Stool microbiome, and in-vitro assays with metformin and SARS-CoV-2
- Cytokine 45-plex assay	- Viral load

NETs, neutrophil-extracellular traps; ACE2 angiotensin-converting enzyme 2; AMPK, AMP-activated protein kinase; GLP=1, glucagon-like peptide-1; IL, interleukin; mTOR, mammalian target of rapamycin.

## Theorized Mechanisms to Support the Use of Metformin in Covid-19

### High Glucose Is Associated With Worse Covid-19 Outcomes

Hyperglycemia was found to portend worse outcomes in severe acute respiratory syndrome (SARS) infection in 2003 (72), and is associated with increased length of stay and mortality in patients hospitalized with Covid-19 (58). Worse glucose control has also been associated with higher mortality and end-organ complications in patients with Covid-19 (15). A recent study by Crouse et al. found that metformin use prior to the diagnosis of COVID-19 was associated with a ~70% decrease in mortality in persons with diabetes (10), though this is a larger effect size than what is seen in most studies (10).

### Metformin Could Decrease Endothelial Injury and Its Complications

Metformin has been shown to improve microvascular endothelial function in women, perhaps due to a significant increase in response to acetylcholine, decrease in insulin resistance, and non-significant decrease in tissue plasminogen activating factor, as was shown in a randomized control study of 8 weeks of metformin *versus* placebo in women with angina and normal coronary arteries (67). Endothelial dysfunction may be an important mechanism and therapeutic target in mitigating Covid-19 sequelae. Metformin decreases thrombosis in long-term follow-up, possibly by inhibiting platelet activation factor and mitochondrial DNA (mtDNA) release (63, 64), and may mediate improved cardiovascular outcomes *via* mechanisms beyond glucose control. COVID associated coagulopathy and thrombosis is a unique attribute of this disease, and the pathophysiology is poorly understood. Widespread micro and macrovascular thrombosis has been reported in autopsies of patients with Covid-19 (65, 66).

### Metformin Has Been Associated With Beneficial Effects on the Lungs

*In vivo* models have demonstrated that metformin overcomes anabolic metabolism promoting AMPK-dependent resolution of lung tissue damage, indicating a potential role in addressing inflammation induced pulmonary fibrosis as a result of severe infections (73). Pulmonary vascular endothelitis has been observed in the lungs of patients with Covid-19 (68). Earlier studies also demonstrated that metformin inhibits AGEs-induced inflammatory response in murine macrophages partly through AMPK activation and RAGE/NF*κ*B pathway suppression, felt to be important in Covid-19 related lung-centric inflammation and endothelitis (74). Further, metformin has been associated with reduced pulmonary fibrosis through reduced TGF-beta and VEGF, and resolution of pulmonary fibrosis *via* activation of lung myofibroblasts (41, 69). Pulmonary fibrosis has been noted in persons with Covid-19, specifically in those with elevated IL-6 levels (70).

### Metformin Has Immune-Modulatory Effects

In patients with and without diabetes, metformin has been shown to favorably alter inflammatory mediators, including interleukin 6 (IL-6), TNF*α*, to possibly boost interleukin 10 (IL-10), and suppress the C-C motif chemokine ligand (34, 44–46). Metformin’s activation of the AMPK/mTor/Stat3 pathway appears to steer macrophages away from the pro-inflammatory classical activation that produces TNF/IL6/IL1b, cytokines that contribute to morbidity in Covid-19 (37, 49, 50). Possible evidence of this effect was seen in a retrospective study by Chen et al: 904 patients with Covid-19 which showed that metformin users had lower IL-6 levels compared to non-metformin users (**Table 2**) (56). Metformin also inhibits toll-like-receptor 7 (TLR7) signaling and interferon production, which appears important to Covid-19 pathophysiology (47, 48). Metformin also inhibits IgE- and aryl hydrocarbon-mediated mast cell activation (59). Mast cell activation has been implicated as an early indicator of inflammatory response to SARS-CoV-2. and possibly an indicator of impending cytokine storm (61, 62). Mast cells from female rats have been found to cause a greater increase in tumor necrosis factor alpha (TNF-alpha) than mast cells in male rats, which may explain the observational findings of reduced mortality in women on metformin, but not among men on metformin (**Table 1**) (60).

Metformin decreases neutrophil-extracellular traps (NETs), and the neutrophil to lymphocyte ratio (34, 51, 52). NETs are microbiocidal compounds containing DNA, histones, and proteins (51, 52). Sera from patients with Covid-19 demonstrate elevated levels of these histones and DNA components (52). It has been hypothesized that excessive NET formation leads to cytokine storm and microthrombus (possibly independent of tissue factor), and ultimately acute respiratory distress syndrome (ARDS) in Covid-19 (54). Lymphopenia and neutrophil infiltration in pulmonary capillaries have been an important feature of severe Covid-19 disease (7, 34, 56, 57). Metformin’s inhibition of NET release could therefore mitigate the development of downstream lung injury.

### Metformin Could Decrease the Viral Cycle

There is some evidence that metformin increases endosomal pH *via* action on vacuolar ATPase and/or endosomal Na+/H+ exchangers (71), thus reducing viral replication. Metformin also leads indirectly to alteration of the mammalian target of rapamycin (mTOR) pathway (38), which could decrease the viral lifecycle through effects on proteins including Orf9c and Nsp7 (38). Orf9c may enable immune evasion (75), and Nsp7 is necessary for RNA polymerase activity (76). Other studies suggest a link between elevated levels of IL-6 and AMPK/mTOR signaling pathway and their role in exacerbating diabetes-induced complications and insulin resistance (77). This has led to mTOR inhibitors being suggested as potential therapeutics in treatment of Covid-19 (78). Gordon et al. did find *in-vitro* efficacy of metformin against SARS-CoV-2, but did not elucidate the mechanism of viral inhibition (79).

### Metformin Could Decrease Entry of the SARS-CoV-2 Into Cells

Activation of AMPK by metformin increases phosphorylation and expression of angiotensin-converting enzyme 2 (ACE2) (80). Phosphorylation of the ACE2 receptor may alter the conformation of the extracellular domain of ACE2 and decrease SARS-Cov-2 entry into cells (6, 39, 42, 43, 81).

Li and colleagues found that expression of ACE2 is equal in male and female human lungs (82), which differs from one prior study showing lower levels of ACE2 receptor in the lungs of middle-aged male rats, however no sex difference in young and old-age rats (83). Li and colleagues did find that the immune response to SARS-CoV-2 in the lungs differs between men and women, with differing cytokine responses (82). This pathologic finding, with our observational findings that metformin conveyed greater protection in women than men, may support that anti-inflammatory effects may be the primary ways in which metformin is protective in Covid-19 (11).

### Metformin Also Reduces Body Weight

Obesity is a known risk factor for poor outcomes in Covid-19 infection, second only to increased age (31). Several studies have shown that metformin use is associated with reduced body weight in patients with and without T2DM (3, 84), and growth/differentiating factor 15 (GDF 15) appears to be an important mechanism of action by which metformin causes weight loss (85). An increase in GDF15 has been associated with decreased food intake and lowered body weight (85). It is unknown if weight loss in the weeks, months, or years prior to a Covid-19 infection would improve outcomes from Covid-19.

## By Improving the Microbiome, Metformin May Limit Systemic Inflammation

In recent years, it has become increasingly clear that healthy microbial communities that make up the microbiome are critical to human health. Obesity, diabetes mellitus, and other metabolic disorders are all associated with imbalanced microbial communities, known as “microbial dysbiosis.” (86) Dysbiosis of the microbiome can result in several deleterious effects that can lead to poor health outcomes, including mucosal and systemic inflammation, microbial translocation, and damage to the tight epithelial barrier of mucosal sites such as the gut. Furthermore, novel studies have demonstrated that Covid-19 disease is associated with microbial dysbiosis, which may be a potential mechanism underlying overt inflammation and dysregulated immunity in Covid-19 disease (87, 88).

Given the above, another mechanism by which metformin may limit disease severity in Covid-19 is by enhancing the microbiome to promote anti-inflammatory effects. Indeed, several studies have demonstrated that metformin can alter the microbiome in a potentially beneficial manner. This includes: (i.) increased “probiotic” strains (i.e. beneficial, anti-inflammatory bacteria such as *Lactobacillus*) (89). (ii.) increased bacteria strains such as *Bifidobacteria, Megasphera, Ruminococcus*, and *Butyrivibrio* that produce short-chain fatty acids (89, 90) which are essential for epithelial barrier function and regulation of inflammation; (iii.) increased bacteria strains such as *Bacteroides* spp. that produce bile acids, which are essential in cholesterol homeostasis and metabolic health (91); (iv.) improvement of microbial communities associated with decreased overall inflammation through lowered TLR-4 signaling, microbial translocation and barrier dysfunction (86, 89); (v.) increased levels of bacteria such as *Akkermansia* species that degrade mucins, which can prevent biofilms from forming that can promote inflammatory bacteria species; and (vi.) reduced bacteria known to be associated with barrier damage and inflammation including *Prevotella.* While there are some conflicting reports regarding microbial alterations after metformin treatment (92), this is likely due to confounders and study design. Overall, it appears that metformin improves the microbiome and can contribute to better mucosal health and overall lowered inflammation. Thus, a potential mechanism by which metformin may improve prognosis in Covid-19 may be through improvement of the microbiome and downstream lowered inflammation.

## Metformin and Sex-Specific Inflammatory and Mortality Findings

As mentioned, observational data suggest a mortality benefit only among female adult patients with diabetes or obesity hospitalized with Covid-19 (**Table 1**) (11). Another study found reduced incidence of ARDS in patients with Covid-19 taking metformin *vs* those who were not; however, this association was only found among females (27). Additionally, metformin use has been associated with a sex-specific mortality benefit in women compared to men with colorectal cancer (23). Possible reasons for sex-specific effects of metformin include the influence of sex hormones and epigenetic changes on the Y chromosome (93). While metformin has been associated with decreased TNF-alpha use after starting metformin in both men and women, these benefits have been shown to be greater in females *versus* males in several studies (see **Table 1** **)** (23, 25, 34, 94, 95). These sex-specific findings may be related to c-src modulation of sex steroids (96).

The National Institutes of Health started to seriously promote sex as a biologic variable in 2014 (97). Much of the mechanistic research into the basic science behind metformin was done before 2014, which may limit the understanding of sex-specific effects of metformin. This remains an important area for future investigation (98).

## Metformin’s History of Anti-Infectious Properties

Metformin was found to have antiviral activity before SARS-CoV-2. In the 1940s and 1950s, metformin was used against influenza (as “Flumamine”), and was found to be effective against parainfluenza and cowpox (1, 99). Metformin has also been associated with improved affect against tuberculosis and is being assessed in HIV (100). With the Zika virus, another RNA virus, activation of AMPK by metformin resulted in restricted viral replication by potentiating innate antiviral responses and decreasing glycolysis, with PKA Inhibitor PKI leading to decreased viral infection and replication (101, 102). Metformin was only assessed *in-vitro* against Zika, not *in-vivo*. In patients with hepatitis C, metformin has been associated with improved virologic response to antivirals and decreased insulin resistance (103). Metformin’s past anti-infectious benefits have been both against the infectious agent directly, as well as by improving the underlying health of the human host. In May 2020, Gordon et al. did find that metformin both reduced SARS-CoV-2 virus and improved cell viability during *in-vitro* assays (79). It is unknown if the lower mortality observed in patients infected with Covid-19 who are on metformin is due to direct activity against the virus itself, improved host substrate (i.e. lower inflammation pre-infection, or lower biologic aging), or both.

## Safety

Metformin is overall a safe medication that has been widely used for decades (104). It is well-tolerated in most individuals, and there is flexibility in the timing of administration which can improve side-effects and adherence (105). Metformin use without other glucose-lowering medications does not lead to glucose reduction below physiologic levels. For this reason, it is considered a safe medication among older adults (106). The most common safety concern is the possibility of lactic acidosis, but this adverse side effect is rare (104) A review found that, even in patients with advanced liver disease, the risk for lactic acidosis is low (107). Recent evidence from Cheng et al. also found that, *inpatient* metformin use of 2 to 3 g/day was significantly associated with an increased incidence of developing lactic acidosis (OR, 22.57; 95% CI, 1.99–256.71; p = 0.012) and acidosis (OR, 12.79; 95% CI, 1.24–132.14; p = 0.032), neither low-dose (<1 g/day) nor moderate-dose (1 to 2 g/day) was significantly associated with the acidosis or lactic acidosis. Additionally, the incidence of heart failure was significantly lower in the metformin group compared to the non-metformin group (adjusted HR, 0.61; 95% CI, 0.43–0.87; p = 0.006) (19). An analysis of individual case safety reports of persons with Covid-19 on hydroxychloroquine suggested an increased risk of mortality associated with hydroxychloroquine + metformin use (ROR value of 57.7 (23.9-139.3) compared to hydroxychloroquine use alone (16). The authors found autophagosomes in mice treated with both, and hypothesized that the excess mortality was from a synergistic inhibition of autophagy (16). Caution should be considered for co-administration in humans.

There is currently a voluntary FDA recall of some long-acting metformin. While some manufacturers of long-acting metformin are currently under voluntary recall because of elevated nitrosodimethylamine (NDMA), a water treatment chemical, no elevated levels of NDMA have been found in the short acting formulation (108).

Additionally, while metformin does cross the placenta, it appears to be safe and has been used off-label in pregnancy. In studies randomizing pregnant women to glucose-lowering therapy, metformin was associated with lower gestational weight gain and a lower risk of pre-eclampsia compared with insulin. Further, other randomized controlled trials have found that metformin is associated with reduced risk of hypertensive disorders of pregnancy in women with obesity or diabetes mellitus (109, 110). Most medications being considered for Covid-19 treatment and prevention are safe during pregnancy.

## Conclusion

The goal of this targeted review was to provide a high-level overview of initial data around metformin and Covid-19, and mechanistic theories and data that pre-dated Covid-19. Observational studies have suggested associations with decreased risk of mortality in patients who were on metformin before being hospitalized with Covid-19. Observational studies are significantly limited by confounding by indication and contraindication. While many laboratory studies suggest plausible mechanistic reasons why metformin would be protective in Covid-19, and metformin has been shown to inhibit SARS-CoV-2 *in vitro*, the data so far are not conclusive. Given the above-mentioned observational findings, plausible mechanisms of metformin’s effect in reducing Covid-19 related morbidity/mortality, acceptable safety profile, low cost, and the devastating nature of the global Covid-19 pandemic, metformin should be prospectively assessed as a potential Covid-19 treatment. Additionally, observational studies should assess outcomes in individuals who are chronically on hydroxychloroquine and metformin, as there may be a safety issue in this combination.

While vaccine development for SARS-CoV-2 has been promising and is the most important approach to preventing severe COVID-19, there may be reduced willingness among the public to receive a vaccine developed so quickly. Additionally, it will be many months before vaccines are available world-wide, some individuals will still get Covid-19 even after vaccination, and at times viral variants may evade vaccine effectiveness until new vaccines can be distributed (111). Because medical practice should not change without being informed by rigorous data, randomized clinical trials should be done to prospectively assess safe and readily available medications such as metformin for reducing the risks associated to Covid-19.

## Author Contributions

SI contributed to the planning and execution of the significant revisions. JL contributed to the updating of the manuscript and content. CB wrote the initial draft of the manuscript. NI contributed to critical review and making the figure. SS contributed to critical review and making the figure. NK wrote parts of the article and contributed to critical review. NS contributed to critical review. LA contributed to critical review. MP contributed to critical review and designing the article. LT contributed to critical review and making the figure. AP contributed to critical review. EB contributed to critical review. MU contributed to critical review and making the figure; BB contributed to critical review. DV contributed to critical review. CT contributed to critical review and conceptualizing the manuscript. All authors contributed to the article and approved the submitted version. 

## Funding

1. This research was supported by the Agency for Healthcare Research and Quality (AHRQ) and Patient-Centered Outcomes Research Institute (PCORI), grant K12HS026379 (CJT).
2. This research was supposed by the Minnesota Learning Health System Mentored Training Program (MH-LHS), M Health Fairview Institutional Funds (CTB, SS).
3. This research was supported by the National Center for Advancing Translational Sciences, grants KL2TR002492 and UL1TR002494 (CTB).
4. This research was supported by the National Heart, Lung, Blood Institute T32HL07741 (NEI).
5. This research was supported by the National Cancer Institute (NCI), grant P30CA077598.
6. This research was supported by COVID-19 Rapid response grant UM 2020-2231.

## Conflict of Interest

DV is employed by the company UnitedHealth Group.

The remaining authors declare that the research was conducted in the absence of any commercial or financial relationships that could be construed as a potential conflict of interest.
